# Behavioral and cognitive animal models in headache research

**DOI:** 10.1186/s10194-019-0963-6

**Published:** 2019-01-31

**Authors:** Doga Vuralli, Anne-Sophie Wattiez, Andrew F. Russo, Hayrunnisa Bolay

**Affiliations:** 1 Department of Neurology and Algology, Gazi University Faculty of Medicine, Besevler, 06510 Ankara, Turkey; 2 Neuropsychiatry Center, Gazi University, Besevler, 06510 Ankara, Turkey; 3 Department of Molecular Physiology and Biophysics, University of Iowa, Iowa City, IA USA; 4 Center for the Prevention and Treatment of Visual Loss, Iowa VA Health Care System, Iowa City, IA USA; 5 Department of Neurology, University of Iowa, Iowa City, IA USA

**Keywords:** Headache models, Migraine, Rodents, Pain behavior, Tactile allodynia, Anxiety-like behaviors, Cognitive assessment in animals

## Abstract

Animal models have provided a growing body of information about the pathophysiology of headaches and novel therapeutic targets. In recent years, experiments in awake animals have gained attention as more relevant headache models. Pain can be assessed in animals using behavioral alterations, which includes sensory-discriminative, affective-emotional and cognitive aspects. Spontaneous behavioral alterations such as increased grooming, freezing, eye blinking, wet dog shake and head shake and decreased locomotion, rearing, food or water consumption observed during pain episodes are oftentimes easy to translate into clinical outcomes, but are giving little information about the localization and modality of the pain. Evoked pain response such as tactile and thermal hypersensitivity measures are less translatable but gives more insight into mechanisms of action. Mechanical allodynia is usually assessed with von Frey monofilaments and dynamic aesthesiometer, and thermal allodynia can be evaluated with acetone evaporation test and Hargreaves’ test in animal models. Anxiety and depression are the most frequent comorbid diseases in headache disorders. Anxiety-like behaviors are evaluated with the open-field, elevated plus-maze or light/dark box tests. Interpretation of the latter test is challenging in migraine models, as presence of photophobia or photosensitivity can also be measured in light/dark boxes. Depressive behavior is assessed with the forced-swim or tail suspension tests. The majority of headache patients complain of cognitive symptoms and migraine is associated with poor cognitive performance in clinic-based studies. Cluster headache and tension type headache patients also exhibit a reversible cognitive dysfunction during the headache attacks. However, only a limited number of animal studies have investigated cognitive aspects of headache disorders, which remains a relatively unexplored aspect of these pathologies. Thus, the headache field has an excellent and growing selection of model systems that are likely to yield exciting advances in the future.

## Introduction

Headache disorders are one of the most common conditions in medicine with an overall prevalence of 48.9% in the general population [1]. Most studies have focused on migraine headache with its characteristic clinical features. Our understanding of headache pathophysiology is mainly based on animal models.

Noxious signals transduced at nociceptors within cranial structures such as dura mater, arteries, scalp and muscles are transmitted centrally to be processed, modified and perceived as headache. The ophthalmic branch of trigeminal nerve and upper cervical nerves play a crucial role in transmission of pain sensation from intracranial structures and converge on second order neurons in the trigeminal spinal tract (trigeminal nucleus caudalis /trigeminocervical junction). In relation to the trigeminal innervation pattern, the dura mater is the intracranial structure sensitive to pain whereas, brain parenchyma is pain insensitive. Upon activation of perivascular trigeminal nerve endings, anti-dromic neuropeptide release induces neurogenic inflammation in the dura mater and alters vascular physiology [2, 3]. Pain and temperature sensation elicited in the dura mater are conducted via Aδ and C fibers of pseudo-unipolar neurons in the trigeminal ganglion to the second order neurons [4, 5]. Trigeminal afferents from spinal trigeminal nucleus to third-order neurons in the thalamic ventroposteromedial nuclei are then projected to the primary and secondary somatosensory cortices. The aforementioned pathway is called the lateral pain system and related to sensory discriminative aspects of headache perception. Second order nociceptive neurons in the caudal brain stem trigeminal tract also project to the anterior cingulate cortex and insula through parabrachial nucleus, amygdala and medial thalamus. The latter pathway is known as medial pain system and plays a role in the emergence of affective aspects associated with headache [4]. The prefrontal and orbitofrontal cortices are involved in cognitive modulation of pain whereas the thalamic reticular nucleus is involved in attention and lateral inhibition processes during headache perception.

Extensive axonal branches of trigeminal nerve within the intra- and extra cerebral structures are important for referral pain [4]. Transmission of noxious signals orthodromically to 2nd order trigeminal neurons, could also activate axonal reflex inducing pain in the referral area. Moreover, the convergence of trigeminal input onto cervical dorsal horn neurons or development of central sensitization provides other mechanisms for reflecting pain outside trigeminal receptive field.

Animal models of migraine and other headache disorders provide a better understanding of the pathophysiology of headache disorders and pharmacological basis for treatment. Since pain experience is subjective by nature and cannot be measured directly, pain in animals is inferred on the basis of pain-like behaviors. Behavioral assays used in animal models are imperative for the correlation of basic research with actual headache experience in humans. Sensory-discriminative, affective-emotional and cognitive aspects of pain can be assessed with specific tests. This review will focus on the behavioral and cognitive assays used in animal models of headache research and we will summarize the behavioral and cognitive models for nociception and associated symptoms.

## Pain behavior

Pain and behavioral outcome are challenging to assess in preclinical rodent studies. Most studies have tried to indirectly assess pain by non-evoked behaviors. This section summarizes the different spontaneous pain behaviors observed in animal models in headache research.

### Assessment of spontaneous behaviors

Natural behaviors in rodents, such as exploratory behavior, locomotor activity, rearing or even food and water consumption, can be decreased during a painful event. Those spontaneous behaviors are therefore assessed in preclinical research as indirect markers for noxious experience.

Locomotor activity has been assessed in several headache models with varying results. Repeated application of inflammatory soup (bradykinin, serotonin, prostaglandin E2 and histamine) onto the dura mater decreased the distance travelled, increased inactivity and reduced exploratory behavior in a dose dependent manner [6]. Locomotion measured over 15 min after inflammatory soup application, showed lower concentrations were required to elicit a response in females than in males [6]. Another study using application of inflammatory soup onto the dura of male rats reported increased resting and decreased exploratory behavior for at least 45 min [7]. These phenotypes were partially attenuated by administration of zolmitriptan (5-HT_1B/D_ agonist anti-migraine drug), ketolorac or acetaminophen prior to dural inflammatory soup exposure [7]. Application of transient receptor potential A1 (TRPA1) agonists such as mustard oil and umbellulone onto the dura mater did not alter the time spent exploring or the total distance travelled [8]. However, a microinjection of the TRPA1 agonist allyl isothiocyanate onto the dura mater was able to significantly decrease the running wheel activity in rats [9]. A reduction in running wheel activity (wheel placed in the home cage) can be a good indicator of voluntary activity, which can reflect the painful state of the animal. The reduced wheel running was concentration dependent upon repeated injections and could be attenuated by sumatriptan (another 5-HT_1B/D_ agonist anti-migraine drug), but only at a high dose and if the drug was injected at the time of induction [9].

Induction of cortical spreading depression (CSD) as a counterpart of migraine aura has also been used as a migraine model in awake rats [3, 10] and recently optogenetic stimulation was used to induce CSD non-invasively in awake, moving mice [11]. In a model of single CSD induced by dural N-methyl-D-aspartate (NMDA) application, decreased locomotor activity was also observed, along with other nociceptive behaviors but did not reach statistical significance [10]. Multiple CSDs induced by administration of potassium chloride (KCl) onto the dura mater significantly reduced total distance travelled [12, 13], and this behavior was partially restored by co-administration of valproic acid [10]. Contrary to results obtained in other models, a single injection of nitroglycerin (NTG) in rats increased locomotor activity compared to control animals [14]. One explanation for this surprising result is the use of saline injection for the control group rather than propylene glycol and ethanol which are used to dilute NTG. In the same study, rats receiving 3 or 5 administrations of NTG showed significantly decreased locomotor activity compared to saline controls [14]. The same group assessed the effect of the propylene glycol and ethanol vehicle in a subsequent study and showed that both the NTG and vehicle groups had decreased activity compared to saline injected group over up to 4 repeated administrations [15]. Only after the fifth administration did the vehicle group cease to differ from the saline group whereas the NTG group still displayed some reduced activity [15]. In an optogenetic study, a transient increase in active behavior alternated with one or more short periods of immobility, similar to freezing-like behavior, was observed 1–3 min after the initiation of CSD induced by optogenetic stimulation and CSD was also shown to disrupt contralateral forepaw wire grabbing transiently coinciding with the CSD wave propagating over M1 cortex [11].

In another model of migraine, a central injection of calcitonin gene-related peptide (CGRP) was reported to increase the time resting in the dark, decrease exploration/distance travelled, time spent moving and the number of transition in between the zones of the light/dark assay in both wild type and *nestin/hRAMP1* mice [16, 17]. *Nestin/hRAMP1* mice are genetically altered animals that overexpress the human receptor activity-modifying protein 1 (RAMP1) subunit of the CGRP receptor in the nervous system [17]. Co-administration of the anti-migraine drug rizatriptan attenuated the effects of CGRP on motility [18]. Consistent with the effect of centrally administered CGRP, peripheral administration of CGRP also reduced motility in the dark zone of the light-aversion assay, increased resting in the dark, and decreased ambulatory distance [19].

Rearings are related to exploratory and motor activity. Reduced rearing behavior is also suggested to reflect a balance or vertiginous problem, which could be of importance in migraine. Application of TRPA1 agonists such as mustard oil and umbellulone on the dura mater decreased the number and time of vertical rearing behavior compared to vehicle treated rats [8]. In the same study, no change in performance on a rotarod test for motor coordination in the rats was observed excluding a balance problem. Therefore rearing cannot be solely employed to evaluate vertiginous problem, in fact it has to be considered as a component of locomotion. Administration of CGRP to both control and *nestin/hRAMP1* mice also caused a significant decrease in rearing compared to vehicle treated animals [16]. This was observed with both centrally and peripherally administered CGRP [19]. Likewise, an epidural administration of CGRP induced a dose-dependent decrease in rearing behavior of rats in which the animals seized the cage with their front paws [20].

Finally, a few studies assessed food and/or water consumption in models of headache. Food and water intake can indicate an overall decrease in well-being, and it can also hint at nausea, although it is impossible to know for sure. After application of KCl or NMDA to the dura inducing a CSD event, no change in the consumption of food or drink was observed in freely moving rats [10, 12]. Very recently, a team showed that repeated peripheral administration of NTG induced a decrease in food intake, starting after the first injection, and slowly decreasing after each of the 5 injections [19]. Of note in this study, the control group was injected with saline rather than with the ethanol/glycol vehicle in which the NTG was diluted, therefore it is impossible to know whether NTG, its vehicle, or the combination of both are responsible for the observed phenotype. As a result, the body weight of those animals also decreased throughout the experiment and compared to saline injection animals [21].

### Assessment of spontaneous nociceptive behaviors

During painful experiences, some animal behaviors are exacerbated as a result of nociception, such as grooming, freezing, head twitch response (wet dog shake/head shake), eye closure or eye blinking. Over the past few years, assessments of spontaneous pain in rodents have been described in detail and are now being used in headache models.

CSD, considered as a pathophysiological correlate of migraine aura, is used as a migraine model. However, it is still a matter of investigation, whether CSD is implicated in migraine with aura only, or in all migraine subtypes. Single CSD or multiple CSDs induced by topical application of NMDA or KCl respectively cause pronounced and reproducible freezing episodes [10, 12, 13]. Freezing is defined as an episode during which the animal “abruptly stopped moving, walking, rearing, grooming or eating and stared at an uncertain spot” [13]. It is possible that decreased locomotor activity/freezing may indicate the electrical silence and CSD propagation over the motor cortex. The latter is not supported by the occurrence of freezing episodes throughout 30 min following a single CSD and not coinciding with the CSD-induced ECoG amplitude suppression. The number and duration of freezing episodes induced by multiple CSDs were significantly reduced by administration of the CGRP receptor antagonist MK-8825 [13] and valproic acid [12]. However, freezing behavior following a single CSD was not significantly reversed by sumatriptan [10]. After an epidural injection of CGRP, rats had increased immobility, with freezing and resting [20]. In mice, there was a significant increase in predator odor-evoked freezing behavior induced by CGRP in both *nestin/hRAMP1* and control mice [16]. Discriminating freezing behavior embedded within immobility, sleeping and resting behavior is a very basic step in the experiments though once detected it seems a reliable behavioral marker for head pain.

Although grooming is a natural behavior in rodents, increased grooming, especially if localized to a specific area (such as the head in migraine models) can indicate increased discomfort/nociception. As such, repeated application of inflammatory soup onto the dura mater induced increased grooming and scratching in an open field setting in a dose-dependent manner [6]. In this experiment, female rats displayed more sensitivity than male rats [6]. Even following a single CSD the total duration of body grooming was increased compared to control groups, but not significantly so [10]. Likewise, multiple CSDs induced by dural KCl application significantly increase the grooming behavior [13]. Other behavioral parameters, such as wet dog shakes and head shakes were significantly increased by CSDs [13]. Increased grooming and twitch response of head and body were partially reversed by CGRP receptor antagonist in a dose dependent manner [13]. In another study using inflammatory soup onto the dura of male rats, ipsilateral hindpaw facial grooming was observed, which was attenuated by zolmitriptan [7]. After an epidural injection of CGRP, rats showed significant decrease in facial grooming within 30 min, but no increase in body grooming was observed [20]. An increase in head-directed wiping and scratching was also observed in mice receiving a dural application of capsaicin along with a mixture of inflammatory mediators [22]. In a transgenic model of familial hemiplegic migraine 1 (FHM1), there was no difference in total grooming behavior between wild type and *CACNA1A* mutant mice over a 2 h observation assay. However the mice also had a greater frequency of head directed strokes, and displayed long, isolated strokes centering on the oculotemporal region of the head. Of note, not all the mutant animals showed laterality in their strokes [23].

A new tool to assess pain in animals is the use of facial signs of discomfort such as eye closure, ear orientation, or nose and cheek bulges to name a few, oftentimes regrouped into grimace scales [24, 25]. Because the eye closure component of the scales carry the most weight in the outcome of the assay [26], a few studies also focus on this action unit only (eye closure, eye blink, squint) to assess nociception. In the transgenic model of FHM1, it was observed that mutant mice had eye blinks associated with a whole-body shuddering behavior and spent most of their time with one eye closed when compared to wild type controls [23]. The same animal model showed an increased grimace score compared to control littermates [24]. The repeated induction of CSD with a KCl pellet onto the dura in mice significantly increased the score for the mouse grimace scale, and was suppressed by a pretreatment with a Panx1 inhibitor carbenoxolone [27]. In two consecutive studies, a team first reported that repeated administration of NTG did not induce grimace in rats compared to saline treated controls [14], before reporting increased facial pain expression and increased orbital tightening 30 min after the fifth injection of nitroglycerin compared to saline control group in the second study [15]. In the latter study, significantly increased grimace and orbital tightening were also observed across five NTG administration sessions compared to its vehicle (30% propylene glycol, 30% 200 proof ethanol in saline) [15].

**Fig. 1:**
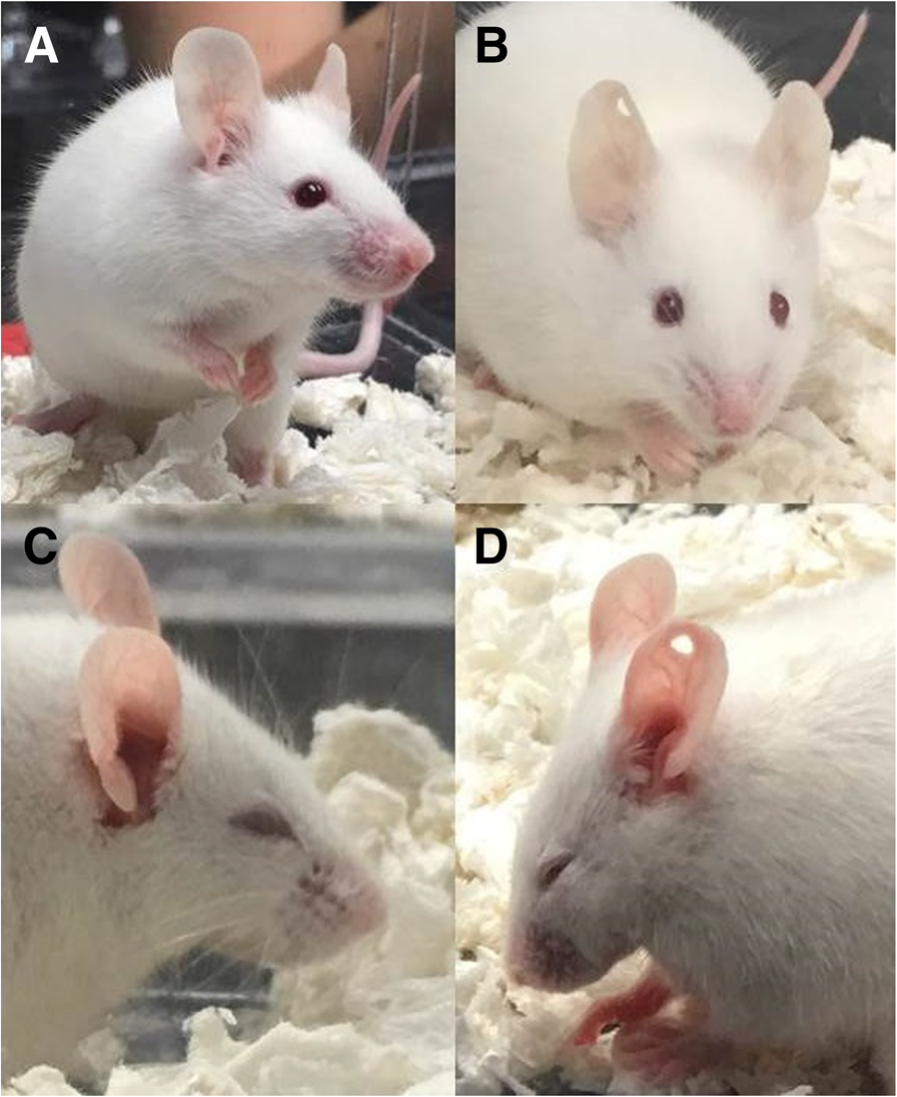
Representative pictures of spontaneous grimace induced in free-moving CD1 mice after peripheral CGRP. **a** Facial features observed at baseline, (**b**) facial features observed 30 min after peripheral administration of saline (PBS), (**c** and **d**) facial features observed 30 min after peripheral administration of CGRP (0.1 mg/kg, i.p)

Recently, pain in a mouse model of migraine induced by peripheral administration of CGRP was described. An increase in facial signs of discomfort using both the mouse grimace scale (Fig. 1) and quantifiable and linear squint assay measuring eye closure in millimeters were defined [26]. The observed phenotypes were reversed by anti-CGRP antibody. Interestingly, sumatriptan partially inhibited CGRP-induced spontaneous pain in males but not females [26].

### Ultrasonic vocalization calls

**Fig. 2:**
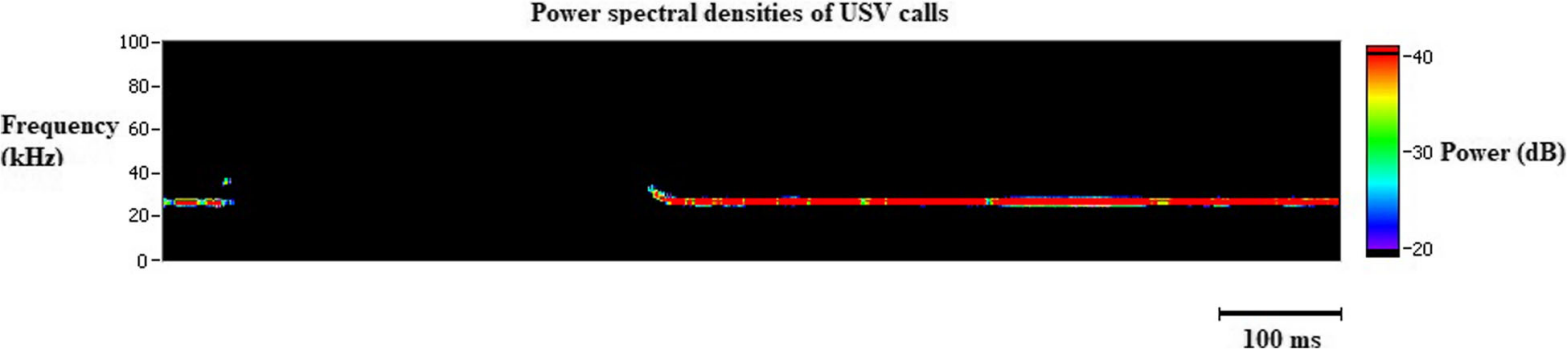
Demonstration of ultrasonic vocalisation (USV) calls in an awake rat following a cortical spreading depression. Emission of USV calls in the 22–27 kHz range in adult rats indicate a negative affective state such as pain or distress. The shift in the frequency at the beginning and at the end of the ultrasonic vocalisation is the typical feature of a biological sound

Among vertebrates, vocalizations are commonly used for communications regarding mother-offspring relations, mating, negative or positive affect (fear, pain, distress or joy), behavioral purposes (approach, avoidance, grooming), presence of predators and location of food. Rats and mice communicate in ultrasonic range (> 20 kHz) [27]. Even though they are inaudible to humans, they can be monitored and analyzed with specialized equipment that uses bandpass filters at 15 kHz and 100 kHz. This removes almost all environmental noises. The 22–27 kHz range of vocalizations (Fig. 2) in juvenile and adult rats indicates a negative affective state seen during pain, distress and exposure to predators whereas 50 kHz vocalizations usually suggests a positive affective state. 5HT1B/1D receptor agonists and non-steroidal anti-inflammatory drugs used in abortive migraine treatment, reduce pain calls in lipopolysaccharide treated rats [28]. Pain calls or stress calls were obtained during freezing episodes of rats that experienced CSD [10].

## Sensory-discriminative aspects

A way to measure pain in animals is to evoke a response to the application of a sensory stimulus. Activation of nociceptive systems alter thresholds in the pain temperature sensation, produce amplified responses to noxious stimulus (hyperalgesia) and/or maladaptive noxious response to innocuous stimuli (allodynia) where both peripheral and central sensitization processes are involved. These features, which are characteristic components of neuropathic pain, can also be seen in migraine patients. Most of the migraine patients complain of cutaneous allodynia in the craniofacial region, which can often extend beyond the trigeminal receptive field to the rest of the body. Moreover, somatosensory temporal discrimination is remarkably prolonged during migraine attacks indicating disruption of sensory stimulus processing. Cutaneous allodynia is more frequent in patients with chronic migraine than episodic migraine, and it correlates with the duration of migraine [29, 30]. Pressure pain thresholds are significantly reduced in both episodic and chronic migraine patients [31, 32].

### Tactile allodynia

There are two types of tactile allodynia; mechanical (pressure) and dynamic cutaneous (brush) allodynia. Mechanical allodynia is evaluated by using von Frey filament application whereas dynamic cutaneous allodynia can be tested via light stroking a paintbrush.

**Fig. 3:**
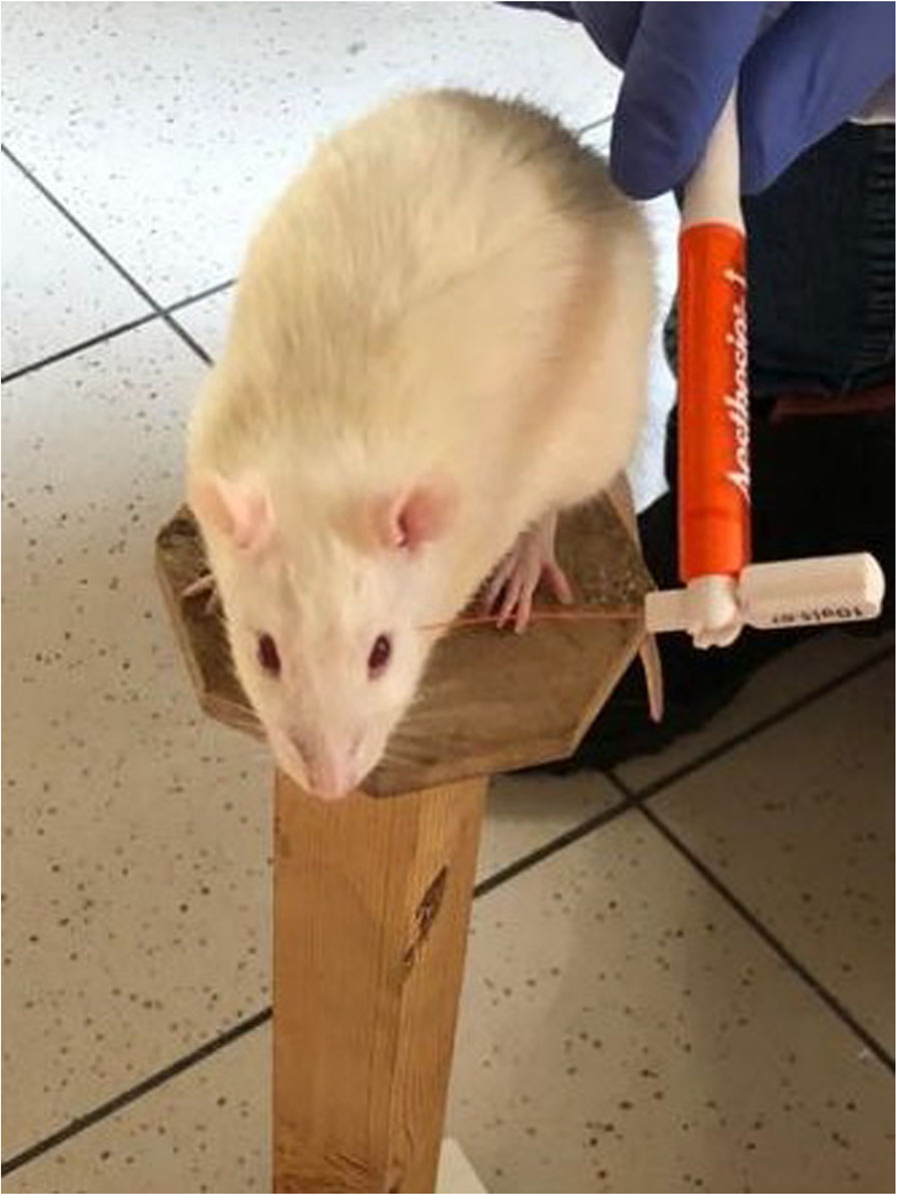
Representative picture of mechanical allodynia assessment using von Frey filaments in a rat. Application of a filament to the periorbital area is demonstrated

The von Frey test, is a method used to evaluate mechanical allodynia in rodents. For the application of von Frey filaments, animals are placed one by one on a small elevated platform (Fig. 3) or in a small cage with a mesh bottom and a monofilament is applied perpendicularly to the periorbital area or plantar surface of the hind paw until it bends. A positive response is brisk paw withdrawal, licking, or shaking of the paw, during application of the monofilament or immediately after the removal of the filament. Different methodological approaches are used such as the “up and down”, “ascending stimulus” and “percent response” methods. In the “up and down” method, testing begins with the monofilament that is the estimated to be close to the 50% withdrawal threshold that evokes a positive response in 50% of animals. If there is no response, testing is continued with a monofilament with a higher force, until a positive response is obtained. When there is a positive response, next lower force filament is tested. Testing is continued until at least four readings are obtained after the directional change and the 50% threshold is calculated. In the “ascending stimulus” method, monofilaments with increasing force are applied until a withdrawal response is evoked, and the force of monofilament that evokes a positive response is recorded as the mechanical withdrawal threshold. In the “percent response” method, von Frey filaments with varying forces are applied in ascending order 5 to 10 times and the number of positive responses to each filament are recorded and then a percent response is calculated.

Multiple studies have shown that single or repeated application of inflammatory mediators directly onto the dura mater elicited both facial and plantar allodynia [6, 33–35], that can be reversed by sumatriptan and the CGRP receptor antagonist CGRP_8–37_ [33]. One study showed that that after a lower dose of dural inflammatory soup, female rats became more responsive to 4 g monofilament than male rats [6]. It was also shown that chronic exposure to inflammatory soup significantly decreased periorbital von Frey thresholds [34]. Direct application of interleukin-6 (IL-6) produced dose-dependent facial and hindpaw allodynia in rats [36]. Interestingly, 72 h after the injection of IL-6, at a time when the animals had recovered from the allodynia symptoms, IL-6-treated rats became sensitive to usually innoxious triggers such as dural application of a pH 6.8 or pH 7.0 solution, or a systemic nitric oxide donor [37]. In the same study, an intracisternal administration of brain-derived neurotrophic factor produced allodynia and primed the rats to subsequent normally innoxious stimulations [37]. Other drugs could also induce allodynia when applied to the dura: TRPA1 agonists such as mustard oil and umbellulone [8], HIV glycoprotein gp120 [35], pH 5.0 synthetic-interstitial fluid [38], and meningeal TRPV4 activators such as a hypotonic solution and 4α-PDD [39].

Mechanical allodynia was not observed following a single CSD induced by pinprick [40], but was present both in the face and hindpaws following multiple CSDs induced by dural KCl application [13, 40]. CSD decreased von Frey thresholds in the ipsilateral periorbital area which was reversed by CGRP receptor antagonist MK-8825 in both 30 mg and 100 mg doses. Consistent with latter finding, CSD induced nociceptive neuronal activation in the trigeminal nucleus caudalis was also significantly suppressed by MK-8825 [13].

Different studies using the NTG-induced model of migraine have reported mechanical allodynia. A single peripheral NTG injection was able to induce mechanical allodynia in mice hindpaw and whisker pads, both of which could be reversed by an injection of sumatriptan [41, 42]. In order to study the progression from acute to chronic migraine, Pradhan and colleagues used chronic injection of NTG every other day for 9 days, which induced progressive and sustained plantar allodynia [21, 43]. This time however, systemic or central sumatriptan did not ameliorate NTG-induced chronic allodynia, but only its acute effect [43]. The application of VL-102 on the dura is also able to activate nitric oxide receptors and evoked acute and chronic cephalic and hindpaw allodynia in a dose-dependent manner [44]. Those phenotypes were blocked by the migraine drugs sumatriptan, propranolol, and topiramate [44]. Intrathecal injection of CGRP induced plantar mechanical allodynia in mice [45], and monofilament response rates were further enhanced in *nestin/hRAMP1* transgenic mice. In those mice, intrathecally administered CGRP also induced a contralateral response after capsaicin injection, which is consistent with central sensitization [45].

Dynamic cutaneous allodynia is assessed via light stroking a paintbrush. In the scoring system of brush allodynia, score 0 is given when there is a very fast lifting of the paw aside. Score 1 is given when there is a prolonged lifting of the paw (> 2 s), score 2 is given when one strong lateral lifting above the level of the body or jumping like a startle reaction is observed and score 3 is given when multiple flinching responses or licking of the stimulated paw is detected. Stimulation with the paintbrush is repeated three times, at least 3 min in-between, and the average of three scores are calculated. Brush allodynia is used in neuropathic pain models, and chronic migraine model developed by nasociliary nerve ligation [46].

### Cold and heat allodynia

#### Acetone evaporation test

The acetone evaporation test is a measure of cold allodynia and measures the aversive behaviors elicited by evaporative cooling [47–49]. Acetone can be dabbed or sprayed on the plantar surface of the hind paw or the periorbital region. Acetone results in a cooling of the skin to the temperatures of 15–21 °C [50, 51], however the temperature can vary depending on the temperature of the room, skin temperature and the amount of acetone applied. When cold allodynia is evaluated in the periorbital region, grooming and avoidance within 1 min is considered as a positive response. The test is repeated 5 times with 5-min intervals, starting from the contralateral side, and positive response is expressed as a percentage. During cold allodynia assessment in the hind paw, acetone is applied alternately three times to each paw and the response to acetone test is scored by the severity of the response (0: no response, 1: quick withdrawal or flick of the paw, 2: prolonged withdrawal or repeated flicking of the paw, 3: repeated flicking of the hind paw and licking of the paw) but the number or duration of nocifensive responses can be also quantified.

Akcali et al. provided a chronic migraine animal model with nasociliary nerve ligation in rats and provoked lateralized headache attack by NTG. In the study, NTG administration increased acetone sensitivity in the forehead only in nasociliary nerve ligated side [46]. In a NTG-induced mouse model of chronic migraine, the duration of withdrawal responses to acetone in the facial region were significantly increased in mice treated with NTG compared to control [52]. Alvarez et al. showed an enhanced response to acetone in neuropathic mice in their model for posttraumatic trigeminal neuropathic pain [53].

#### Hargreaves’ test

Hargreaves’ test is used to evaluate thermal hyperalgesia in unrestrained awake animals. Rats or mice are placed in a plexiglass cubicle with glass floor and a movable infrared source provides thermal stimulus to the plantar surface of the hind paws or tail and when the animal feels pain and withdraws hind paw or tail, the heat source turns off and the reaction time counter stops and the latency to withdrawal from the thermal stimulus is determined. Hind paw has an advantage of independent assessment of either side of the body.

Bates et al. used the Hargreaves’ assay to evaluate thermal nociceptive thresholds after the administration of NTG to determine whether the antimigraine drug, sumatriptan could reverse NTG-induced thermal hypersensitivity. Sumatriptan or saline was injected 5 min after NTG administration. Reduced withdrawal latencies induced by NTG injection were returned to baseline by sumatriptan [41].

### Dynamic plantar aesthesiometer

Dynamic plantar aesthesiometer is used to assess mechanical allodynia. Dynamic plantar aesthesiometer has a metal filament unit 0.5 mm in diameter that raises until it reaches the plantar surface of the hindpaw of the animals that are placed in enclosures on an elevated wire mesh floor and exerts an upwards force until the animal withdraws its paw. The force required to cause a withdrawal response is the mechanical response threshold and is measured in grams.

Sisignano et al. showed that 5, 6-epoxyeicosatrienoic acid synthesized upon acute activation of nociceptors needed TRPA1 to produce mechanical hypersensitivity which was assessed via dynamic plantar aesthesiometer [54]. In a neuropathic pain model achieved by ligating L5–6 spinal nerves, the effect of percutaneous pulsed radiofrequency (PRF) on mechanical allodynia was evaluated and paw withdrawal thresholds were measured via dynamic plantar aesthesiometer [55]. In PRF 2 min group paw withdrawal thresholds were significantly higher than placebo PRF 2 min group on post PRF day 10 [55].

Due to central sensitization, in headache patients, allodynia can extend beyond cranial structures. However, there is no study yet that evaluated extracephalic mechanical allodynia via dynamic plantar aesthesiometer in headache animal models.

## Affective and emotional aspects

Psychiatric disorders such as anxiety and depression are common in headache patients. Approximately half of the migraine patients experience anxiety and 20% experience depression [56]. Open-field, elevated plus-maze or light/dark box tests are used to evaluate anxiety-like behaviors in animals. The forced-swim or tail suspension tests are gold-standard tests for assessing depression and anti-depressant activity of medications. Conditioned place preference test is employed to observe pain (aversive) or pain relief (rewarding) effects of drugs.

### Assessment of general well-being

Home-cage monitoring can provide an insight into the wellbeing of an animal. Home cage locomotor activity, exploratory locomotion, spontaneous burrowing behavior and voluntary wheel running [57] can be used to assess general well-being of an animal. During chronic pain, deficits in rat home cage locomotor activity have been reported [58, 59]. The driving force of exploratory locomotion is different from home cage locomotor activity. Exploratory locomotor activity is driven by the novelty of an environment. In rats, chemical stimulation of the dura, as a preclinical model of headache, decreases exploratory locomotion [7, 8].

Burrowing is another innate behavior which decreases during chronic pain states and can be reversed by analgesic drugs [60, 61]. Wheel running is a motivated locomotor activity and measures voluntary physical activity in rodents. Microinjection of TRPA1 agonist allyl isothiocyanate (AITC) onto the dura has been used to generate migraine-like pain in rats [8]. Activation of dural afferents by AITC produces depression of home cage wheel running that is reversed by sumatriptan [9], Δ9-tetrahydrocannabinol or morphine [62]. However, medication overuse headache following repeated morphine, prolongs the duration of AITC induced depression of home cage wheel running.

### Anxiety-like behaviors

#### Open-field test

Open-field test was originally developed for evaluating emotionality in rodents [63] and it is composed of a wall enclosed (walls high enough to prevent escaping) circular, square or rectangular, unfamiliar area which is large enough based on the animal tested to provide a feeling of openness in the center of the maze. Different types of behavior can be scored, such as ambulation, exploration, latency, rearing, location within the field and escape attempts. Especially the inner zone distance percentage (ID%) and the percentage of inner zone time (IT%) are used to evaluate anxiety. ID% is calculated as the inner zone distance/total distance × 100 and IT% is calculated as the time in the inner zone/300 s × 100. Anxious rodents are afraid to explore and prefer to stay in a safer place, which is the outer perimeter of the open field therefore ID% and IT% are lower in anxious rats.

Open field test often is used to assess anxiety, exploration and locomotion. The test is based on the aversions of rodents to novel, brightly lit, open environments. Bogdanov et al. used open field test to assess the correlation between susceptibility to CSD, the most likely cause of migraine aura, and anxiety and found that increased anxiety-like behavior was correlated with higher frequency of CSDs [64].

In a classical chronic migraine rat model with repeated infusion of inflammatory soup to dura mater, ID% was significantly lower in the inflammatory soup group compared to control group suggesting anxiety-like behavior in inflammatory soup group [65].

#### Elevated plus-maze test

Elevated plus maze (EPM) which was first described by Pellow et al. [66], is a simple and reliable method in rodents to assess anxiety like responses. In EPM test, the maze consists 2 open and two closed arms and the maze is elevated approximately 50 cm from the ground. The rodents are placed in the center of the maze, facing the same closed arm, and the spontaneous behaviors are recorded for 5 min with a video-camera system placed above the maze. The elevated plus maze must be cleaned thoroughly with 70% ethanol after each animal. Rodents must be subjected to EPM test only once. The percentages of closed and open arm entries and the percentages of duration spent in closed and open arms are calculated. Anxious animals tend to stay in the closed arms of the elevated plus-maze.

Filiz et al. [13] investigated the effects of CGRP receptor antagonist (MK-8825) on anxiety responses induced by CSD using EPM test. Though the total duration spent in the closed arms enhanced by CSD in all groups, neither dose of MK-8825 reversed EPM results [13]. CSD induced neuronal activation in the amygdala was also not reversed by CGRP receptor antagonist MK-8825, confirming these behavioral results [13].

In a chronic migraine animal model, the percentage of open arm entries was significantly lower in chronic migraine group compared to controls which supported increased anxiety-like behavior [65].

#### Light/dark box

Light/dark box test is based on innate aversion of rodents for bright light and their tendency to show exploratory behavior in response to new environments. The typical light/dark box has two compartments connected to each other with an opening. The rodent is usually placed in the light chamber first and the behavior of the animal is recorded over a 5–10 min period. The chamber must be cleaned with 70% ethanol between testing each animal. The latency of the first entry into the dark compartment, the percentage of time spent in the light and dark compartments and the number of dark to light transitions are quantified. The interpretation of light/dark box test results is challenging in animal models of migraine since presence of photophobia and/or anxiety would yield a similar outcome.

In an experimental chronic migraine model induced by intermittent intraperitoneal injection of NTG in which the effect of chronic ghrelin treatment on endogenous pituitary adenylate cyclase-activating polypeptide (PACAP) and associated symptoms of migraine was investigated, photophobia and anxiety-like behaviors were determined by the modified EPM and the light/dark box tests [67]. The light/dark box revealed that it took a shorter time for NTG group to enter the dark compartment for the first time and NTG group had fewer transitions between two sides than the vehicle group. In addition, NTG group spent significantly less time in the light side and re-entered the light chamber with a longer latency period after the first entry to the dark chamber. In NTG + ghrelin group, significantly increased total time spent in the light box, transition numbers, latency to enter the dark box for the first time, and decreased latency to re-enter the light box were observed. In this study, NTG group exhibited anxiety-like behaviors and NTG + ghrelin group displayed less anxiety-like behaviors in both modified EPM and light/dark box tests [67].

### Depression

#### Forced-swim test

Forced-swim test (FST) was originally reported by Porsolt et al. [68] and since then it has been the most widely used model for evaluating depression and anti-depressant activity in rodents. Porsolt et al. used a vertical plexiglass cylinder (height 40 cm and diameter 18 cm) which contained 15 cm water maintained at 25 °C. In the pretest (habituation), the rat was placed into the cylinder and allowed to swim for 15 min and then removed from the water, allowed to dry for 15 min in a heated place (32 °C) and returned to its cage. The same procedure was repeated 24 h later in the test session. However this time in the cylinder rat stayed for 5 min and total duration of immobility was measured. The rat was counted as immobile whenever it remained floating passively without struggling in an upright position but slightly hunched, keeping its head just above the water.

In one study that compared the behavioral consequences of chronic headache and chronic mild stress in rats, FST was used to observe depressive-like behavior [69]. No significant difference among groups was found regarding the mean duration of climbing, swimming and immobility behavior in FST [69].

#### Tail suspension test

Tail suspension test (TST) is a model of depressive-like behavior and immobility in the TST shows behavioral despair in a stressful situation. The animal is suspended above the ground by its tail with a tape for 6 min and recorded with a video. Latency to immobility and total immobility time for each one minute block are scored. Animals are considered immobile only when they stay motionless for at least 2 s.

Anti-depressant effect of flunarizine, an anti-migraine prophylactic drug, was investigated using TST in rats and mean duration of immobility was found to be significantly reduced with flunarizine compared to its vehicle [70].

### Aversive conditioning

#### Conditioned place preference test

The conditioned place preference test is a behavioral model used to study the rewarding and aversive effects of drugs. This test is based on the association of a particular environment with a specific drug, followed by the association of a different environment with the absence of the drug (the vehicle of the drug). During training, the animal is given an injection of a drug with potentially rewarding or aversive effects, and then placed into one of the compartments for 30–60 min. On the following day, the rat is injected with the vehicle of the drug and then placed in the other compartment. On alternating days the animal receives the drug and its vehicle for a total of 2 or 3 days each. After the conditioning sessions, a 15 min test session is conducted in which the animal is placed in the center with the gates of the both compartments open and the time the animal spends in each compartment is recorded. If the animals spend significantly more time in the drug-paired compartment, it is defined as conditioned place preference. On contrary, if the animals spend significantly more time in the vehicle-paired compartment, then it is defined as conditioned place aversion.

Relief of pain is rewarding and the animals seek relief. Pain relief-induced conditioned place preference can be used to unmask the pain animal suffers [71–74] and this test can be used to evaluate the effect of migraine medications such as sumatriptan [75].

## Cognitive assessment

Migraineurs often complain of cognitive impairment, particularly deficits in attention and memory. Cognitive symptoms may develop during the premonitory phase and persist throughout the headache phase into the postdrome. Some migraine patients also complain of cognitive dysfunction outside migraine attacks. Migraine attacks are associated with poor cognitive performance in standardized neuropsychological tests consistent with cognitive difficulties subjectively reported during attacks [76]. Majority of clinical-based studies also reveal cognitive impairment during interictal period [76]. Neurophysiological, neuroimaging and clinical pharmacological studies support the symptoms of cognitive dysfunction in migraine. Cluster headache and tension type headache patients also display a reversible cognitive decline during the headache attacks [76]. However, there are only a few animal studies that investigated cognitive aspects of headache.

### Morris water maze

Several water mazes have been developed to assess spatial or place learning and memory, but the one that is referred to as ‘the water maze’ is the Morris water maze (MWM). In the MWM, rats are placed in a large circular pool of water in which there is a hidden platform located in the middle of one of the four quadrants. The platform is invisible because it is beneath the water surface and the water is opaque. Normal rodents quickly learn to swim directly towards the platform from any point at the perimeter of the pool. Rats escape to the platform by learning the spatial position of the platform relative to distal cues. Several measures are evaluated such as swim-path length, latency to find the platform (escape latency) and in each trial, length of the swim path and time spent in the platform quadrant. Rodents rapidly learn to locate an object without seeing, hearing or smelling the object if it remains in a fixed spatial position relative to distal cues [77, 78].

Dilekoz et al. [79], used the Morris water maze to assess spatial learning and memory in FHM1 mutant mice and wild type (WT) mice and found that the time to reach the hidden platform was similar between WT and homozygous R192Q mice during the first training session and gradually decreased in both groups in the following sessions. The rate of decrease in time to find the hidden platform was slower in R192Q mice compared to WT mice, which is consistent with impaired spatial learning in FHM1 mice.

### Attentional set-shifting test

The attentional set shifting task (ASST) measures attention, reversal learning and cognitive flexibility in rats. Animals are trained to dig in bowls filled with bedding to retrieve food reward [80]. In the digging bowls used in simple discrimination, only one of the two dimensions (odor or media) differs whereas compound discrimination introduces the second dimension, but the relevant stimulus in the simple discrimination test still identifies the correct bowl. In the reversals, the animals have to learn that previously correct stimulus is incorrect now and that they have to respond to a previously irrelevant stimulus. For the intradimensional and extradimensional shifts, new exemplars of both the relevant and irrelevant dimensions are introduced and for the extradimensional shift, the previously relevant dimension is changed to the irrelevant dimension. At each stage, 6 consecutive correct responses are required to move to the next stage. Trials to criterion**,** errors and mean correct latency are recorded.

Lesion studies in animals have shown that certain regions of the prefrontal cortex are associated with particular stages of ASST. Lesions of the orbitofrontal cortex lead to deficits in reversal learning whereas lesions of the medial prefrontal cortex interferes with the ED shift performance.

Chronic pain has been shown to be accompanied by cognitive dysfunction. In two previous studies that used unilateral spared nerve injury (SNI) neuropathy as a model of neuropathic pain, ASST was used evaluate attention, reversal learning and cognitive flexibility and right sided SNI was associated with impaired reversal learning. These animals required significantly higher number of trials to successfully terminate the reversal steps of ASST compared to sham and left sided SNI animals, however extra- and intradimensional shift performance remained normal.

There is no preclinical study that used ASST to assess attention, learning and cognitive flexibility in migraine animal models. However ASST is an analogue of the human Wisconsin card sorting task (WCST) and Camarda et al. [81] used WCST to compare the executive functions of migraine patients during interictal period and healthy controls. Migraine patients performed worse than healthy controls without migraine in WCST suggesting an impairment in executive functions in migraine patients outside the attacks.

### The novel object recognition test

The novel object recognition (NOR) test is a simple, quick and pure recognition memory test which is based on the assumption that entering into a new environment or encountering a new object, can evoke approach behaviors in animals. Unconditioned preference for novel objects in rodents is stronger during the first 2 min. In the NOR test, the ability of the animals to recognize a new object in a familiar environment is evaluated. The NOR test has usually two trials. In the first trial, the animal is introduced to the sample object (one or two identical objects) and then the animal is returned to his cage for a retention time. After the retention time, the animal is brought to the testing area and exposed to a familiar (sample object) and a novel object in the second trial. If the animal remembers the familiar object, it will explore the new object more than the familiar one.

In a study that compared attention and memory in FHM1 mutant mice and WT mice using the novel object recognition test [79], the time spent to explore a novel object compared to a familiar object was comparable between WT and R192Q mice whereas, heterozygous S218 L mice performed worse than WT mice, and homozygous S218 L mice performed worse than both WT and R192Q mice. R192Q mutation is a weaker gain-of-function CaV2.1 mutation and is associated with a milder FHM phenotype in patients and in transgenic mice compared to S218 L mutation [82–84]. Distributed learning and memory seems to require more prominent changes in glutamatergic neurotransmission caused by the S218 L mutation [85, 86].

### Five-choice serial reaction time task (5-CSRTT) to evaluate sustained attention

The test consists of a chamber with a wall that contains five nose-poke holes and an opposite wall that has a food tray. Holes can be individually illuminated and have infrared detectors which control the delivery of food pellets. Every time the rat pokes its nose into one of the five holes, a food pellet is delivered into the food tray. The first phase of training period consists of one 30-min session for each day and continues until the rat earns 100 food pellets during the 30-min period. The second phase of training period consists of sessions in which one of the holes is illuminated in a pseudorandom order and when the rat pokes into the illuminated hole, the light turns off, a food pellet is delivered into the food tray and a new trial begins with the illumination of another hole after a 5-s intertrial interval. Phase 2 continues until the rat earns 100 pellets during a 30-min session. The last phase of training period comprises of trials in which a hole is illuminated for 16 s until a response is obtained. If the rat pokes into the illuminated hole within the first 18 s of the trial, it is the correct response and results in the delivery of a food pellet. Incorrect responses and omissions result in a 5-s “time out” period in which lights are turned off. Nose pokes during the time out or intertrial intervals result in another time out. After nine 30 min sessions (or sessions in which rat earns 100 food pellets), the duration of the hole illumination decreases until it reaches 1 s. The test sessions are similar to training sessions. Correct responses require to maintain a sustained attention and the response accuracy is an index of attention.

A common cognitive domain affected in chronic pain is attention. Sustained attention was assessed by 5-CSRTT in rats before and after chronic pain induced by intra-articular injection of Complete Freund’s adjuvant to develop monoarthritis [87]. Persistent pain was shown to be associated with more errors in accuracy and increased omissions in the task trials.

Clinical studies have shown a moderate to marked impairment in sustained attention in migraine patients however there is no animal study that used 5-CSRTT to evaluate sustained attention in an animal model of migraine.

### Contextual and cued fear conditioning test

The contextual and cued fear conditioning test evaluates associative fear learning and memory in rodents. For training, animals are placed in a conditioning chamber and a pair of a conditioned stimulus (an auditory cue) and an aversive unconditioned stimulus (an electric foot shock) is given. On the test day, the animals are placed in the same conditioning chamber and a differently shaped chamber and they are exposed to the same auditory cue. The animals learn and remember an association between environmental cues and aversive experiences and respond to the fear-producing stimulus by displaying freezing behavior. Freezing behavior is a common response to fearful conditions and is considered as an index of fear learning and memory. This test requires 5–10 min/day per animal for 2 days. Automated measurement systems are used to conduct the behavioral analysis.

In one previous study, contextual hippocampal learning was evaluated in FHM1 mutant mice and WT mice with the contextual fear-conditioning test [79]. Basal freezing time was similar between WT and FHM1 mice. Twenty-four hours later, contextual freezing was significantly shorter in R192Q mice suggesting an impairment in contextual hippocampal learning.

## Behavioral models of associated symptoms

### Photophobia

Photophobia or photosensitivity is an altered perception of light that commonly triggers migraine, and also elicits discomfort between headaches [88–93]. In animals, light-aversion is used as a surrogate for photophobia. The traditional way to assess photophobia is to use the light/dark box, which consists in two juxtaposed equally sized compartments, one not lit (dark box), and one for which the light intensity is variable from very dim to very bright light (light box) [18]. The animals are tracked by video or infra-red beams to determine time spent in each compartment, as well as motility, rearing and transitions (see chapter 2a). A mouse that spends less time in the lit compartment than control animals is light-aversive. Additionally, since light is naturally anxiogenic for nocturnal rodents used in preclinical settings, this assay needs to be coupled with an independent, non-light dependent measure of anxiety (such as the open-field test) in order to differentiate light-aversion from anxiety.

Using this method, light-aversion was assessed in different animal models of headache. Administration of both central and peripheral CGRP in both CD1 and C57BL/6J mice induced light-aversion to very bright light, while it did not induce anxiety in the open-field test [18, 19]. This phenotype was attenuated by sumatriptan and an anti-CGRP antibody [19]. CGRP also caused transgenic *nestin/hRAMP1* mice to spend less time in the light than control littermates even with very dim light, while performing similarly in the open field, indicating that those CGRP sensitized mice are light-aversive [17]. These results suggest that CGRP actions to induce light-aversion are mediated by both peripheral and central mechanisms [19].

Using the same paradigm, results obtained with NTG are controversial. An infusion of a low dose of NTG in rats was not able to consistently induce light-aversion [94]. In a different study, a single peripheral administration of NTG induced photophobia both in the early (0–30 min) and late phases (90–120 min) after injection [95]. Those phenotypes were significantly reduced in transgenic animals lacking pituitary adenylate cyclase-activating polypeptide [95]. In another study, up to 4 administrations of NTG were not sufficient to induce light-aversion in rats exposed to 260 lm, however, after the fifth administration over a 2 week period, the NTG group showed a significant decrease in the time spent in the light chamber compared to the saline group but not the vehicle group [15]. Similarly, another study showed that NTG injection induced the same amount of light aversion that its vehicle injection [42]. In a very recent study and using a similar automated place preference assay, female rats injected with inflammatory soup onto the dura displayed photophobia to a 250 lx light stimulus, and phonophobia to a 75 dB white noise [96]. Those two phenotypes were observed after up to 7 applications of the inflammatory soup [96].

While photophobia is traditionally assessed using the light/dark box exploratory test described above, it can also be measured using a modified EPM assay [22]. In this test, the EPM is repurposed to create a conflict between anxiety and light-aversion, with the closed arms (safe environment) illuminated with very bright lights, and the open arms (anxiogenic environment) in the dark. During the normal EPM assay, mice would spend more time in the closed arms than in the open arms (see Chapter 4b). Here though, if mice develop enough light-aversion, they might choose to spend more time in the open arms that are dark rather than in the closed arms that are bright. This was observed with the FHM1 mouse model, which spent more time in the dark open arms of the maze than wild type mice used as controls [22]. Female mice tended to spend even more time in the open arms than males, but this didn’t reach significance.

Using both assays, repeated administration of NTG in rats induced light-aversion compared to saline-injected animals, as showed by a shorter latency to enter the dark box, a smaller number of transitions during the assay, a decreased time spent in the light, a longer latency to re-enter the light box, an increased time spent in the dark open arms and an increased number of entries in the dark open arms [67]. Once again, it should be noted that the effect of the vehicle has not been assessed in this study, making it difficult to conclude that NTG is responsible for this effect.

### Nausea and vomiting

Migraine has associated symptoms such as nausea and vomiting however, rats and mice are incapable of vomiting. Therefore, as an alternative to studies of vomiting, conditioned taste aversion paradigms are used in rats, in which they learn to avoid the taste that is paired with toxins causing nausea [97] Loss of appetite is another finding associated with pain and rats show loss of appetite after activation of the trigeminovascular system by dural administration of inflammatory soup.

## Conclusion

Animal models have enhanced our knowledge about the pathophysiology of headaches, especially migraine. These models have been crucial in the development of new therapeutic targets. Animals subjected to pain stimuli will change their behaviors. Different aspects of pain such as sensory-discriminative, affective-emotional and cognitive aspects can be assessed with specific behavioral assays. Some behaviors are directly related to nociception, such as freezing, grooming, or eye blinking. These behaviors can develop or be exacerbated during a painful episode while other behaviors such as locomotor activity, rearing, food or water consumption can be decreased. Activation of pain systems also results in enhanced sensitivity to innoxious stimuli and migraine patients complain of both cranial and extracranial allodynia. Mechanical and thermal allodynia and the impact of therapeutics on allodynia can be evaluated with various methods in animal models of migraine. Anxiety and depression are common comorbidities in migraine patients. The open-field, elevated plus-maze or light/dark box tests are used to evaluate anxiety-like behaviors in animals and the forced-swim or tail suspension tests are used to assess depression and anti-depressant activity of medications. Migraine, cluster headache and tension type headache attacks are associated with poor cognitive performance in clinic-based studies, consistent with cognitive complaints of patients. However, there are only limited number of animal studies that investigated cognitive aspect of headache. Behavioral and cognitive assays used in headache animal models could provide new information about pain pathways and novel targets for headache treatment. However, since it has always been a challenge to interpret certain behavioral alterations in rodents as expression of pain, behavioral testing should not stand alone but be combined with supportive approaches such as biochemistry, pharmacology or histochemistry.

## Abbreviations

5-CSRTT: 5-choice serial reaction time task
AITC: Allyl isothiocyanate
ASST: Attentional set shifting task
CGRP: Calcitonin gene-related peptide
CSD: Cortical spreading depression
EPM: Elevated plus maze
FHM1: Familial hemiplegic migraine 1
FST: Forced swim test
ID%: Inner zone distance percentage
IT%: Percentage of inner zone time
KCl: Potassium chloride
MWM: Morris water maze
NMDA: N-methyl-D-aspartate
NOR: Novel object recognition
NTG: Nitroglycerin
PRF: Pulsed radiofrequency
RAMP1: Receptor activity-modifying protein 1
SNI: Spared nerve injury
TRPA1: Transient receptor potential A1
TST: Tail suspension test
WCST: Wisconsin card sorting task
WT: Wild type
